# Identification of resistance gene analogs of the NBS-LRR family through transcriptome probing and *in silico* prediction of the expressome of *Dalbergia sissoo* under dieback disease stress

**DOI:** 10.3389/fgene.2022.1036029

**Published:** 2022-10-07

**Authors:** Siddra Ijaz, Imran Ul Haq, Iqrar Ahmad Khan, Hayssam M. Ali, Sukhwinder Kaur, Hafiza Arooj Razzaq

**Affiliations:** ^1^ Centre of Agricultural Biochemistry and Biotechnology, University of Agriculture Faisalabad, Faisalabad, Pakistan; ^2^ Department of Plant Pathology, University of Agriculture Faisalabad, Faisalabad, Pakistan; ^3^ Institute of Horticultural Sciences, University of Agriculture Faisalabad, Faisalabad, Pakistan; ^4^ Botany and Microbiology Department, College of Science, King Saud University, Riyadh, Saudi Arabia; ^5^ Department of Plant Pathology, University of California, Davis, Davis, CA, United States

**Keywords:** *Dalbergia sissoo*, resistance gene analogs, NBS-LRR, functional annotation, computational biology

## Abstract

*Dalbergia sissoo* is an important timber tree, and dieback disease poses a dire threat to it toward extinction. The genomic record of *D. sissoo* is not available yet on any database; that is why it is challenging to probe the genetic elements involved in stress resistance. Hence, we attempted to unlock the genetics involved in dieback resistance through probing the NBS-LRR family, linked with mostly disease resistance in plants. We analyzed the transcriptome of *D. sissoo* under dieback challenge through DOP-rtPCR analysis using degenerate primers from conserved regions of NBS domain-encoded gene sequences. The differentially expressed gene sequences were sequenced and *in silico* characterized for predicting the expressome that contributes resistance to *D. sissoo* against dieback. The molecular and bioinformatic analyses predicted the presence of motifs including ATP/GTP-binding site motif A (P-loop NTPase domain), GLPL domain, casein kinase II phosphorylation site, and N-myristoylation site that are the attributes of proteins encoded by disease resistance genes. The physicochemical characteristics of identified resistance gene analogs, subcellular localization, predicted protein fingerprints, *in silico* functional annotation, and predicted protein structure proved their role in disease and stress resistance.

## 1 Introduction

*Dalbergia sissoo* is an important perennial tree of great economic importance due to its significance in agroforestry, forestry, and horticulture. Although it is native to the sub-Himalayan tract, it is abundantly found in other regions of Asia and the southern and central countries of America as well (Ijaz and Haq, 2021). Its high-quality timber is used in furniture making, fuel, etc. It had been severely affected by dieback disease, which was announced as an epidemic for this tree, mostly in the Punjab province (Haq et al., 2021; Ijaz and Haq, 2021). Since then, dieback losses in the *D. sissoo* trees have increased by 40–80%. More than 70% of plant diseases are caused by fungi (Moore et al., 2011; Ul Haq et al., 2022). Plant scientists have documented the strong involvement of phytofungi in trees’ decline compared to other ecological dynamics, including edaphic, climatic, and biotic factors. The decline is the most threatening stress on tree species, and the *D. sissoo* decline leads this tree to the danger of extinction (Orwa et al., 2009; Shah et al., 2010). The use of resistant germplasm to control dieback will be the most effective way to attain the disease’s sustainable management, especially for new plantations (Minocha et al., 2000). Plants possess complex defense mechanisms against phytopathogens (Sarwar et al., 2022a) that lead to biochemical and physiological alterations in plants. The first level includes pathogen recognition, anti-pathogenic protein production, interruption of pathogen infection structures, and enzymatic cell wall reinforcement (Sarwar et al., 2022b). If a pathogen overcomes it, then the second level is initiated. This level includes resistance (R) genes or their products and starts a molecular cascade of signal transductions in response to the attack (Wang et al., 2015), including kinases, phytoalexin, peroxidases, reactive oxygen species, and guanine nucleotide-binding proteins (Meng and Zhang, 2013; Zhong et al., 2019; Çetinel et al., 2022).

Resistance (R) genes encode the effector-triggered immunity system (Jones and Dangl, 2006). Most R genes encode intracellular proteins and have nucleotide-binding site (NBS) and leucine-rich repeat (LRR) domains. These proteins belong to the apoptotic ATPase (AP-ATPase or NB-ARC ATPase) family of STAND (Signal Transduction ATPase with numerous domains) P-loop NTPase. These NTPases are signal-generating bodies and act as plant and animal defense mechanisms’ switches. In the NBS-LRR class of proteins, apart from the NBS and LRR domains, a homologous region is present between these two domains, known as ARC (Riedl et al., 2005). Multiple conserved motifs, including P-loop, kinases, hydrophobic GLPL, and RNBS (resistance nucleotide-binding site), are present in the NB-ARC domain of R proteins (Baldi et al., 2004). A total of three peptide motifs in NBS are crucial for nucleotide-binding in various ATP/GTP bindings. These peptides include the P-loop (also known as a kinase-1a motif or Walker A motif), kinase-2 motif, and kinase-3a motif (Babar et al., 2021; Nasir et al., 2021).

Because of the plant defense system against pathogens and R genes’ involvement in disease resistance, it is crystal clear that identifying plant disease-resistant genes helps disease-resistant breeding in plants and provides insight into the resistance mechanism. Therefore, considering these perspectives, the *D. sissoo* transcriptome was probed to identify the genetic elements differentially expressed under the dieback challenge. This research study was conducted under the CAS-PARB project no. 952. In this study, we attempted to explore *D. sissoo*’s transcriptome through a degenerate oligonucleotide-primed polymerase chain reaction because of the unavailability of its genomic data to identify resistance genes that contribute to dieback disease resistance. Differentially expressed gene sequences (under the dieback challenge) were identified and characterized using bioinformatics tools. This study paves the way toward resistance gene identification in *D. sissoo* (genomic record not available yet on any database) as the contributors of disease-resistance pathways that switch on under the dieback challenge.

## 2 Materials and methods

### 2.1 Plant material collection, plant inoculation, and screening

We collected two hundred plants of *Dalbergia sissoo* across Pakistan. The collected plant material maintained in the greenhouse was macro propagated and screened against the pathogen of dieback diseases. Macro propagated tagged material *of D. sissoo* was inoculated with the fungal pathogen *Ceratocystis dalbergiae* (MycoBank 841380). The inoculation was done by mixing fungal isolate into the soil of growing *D. sissoo* plants.

### 2.2 Genomic DNA extraction

Before plant inoculation, we extracted their total genomic DNA using a eukaryotic genomic DNA extraction kit (Thermo Scientific, United States) and analyzed it through PCR based on a 16S DNA marker for bacteria and an ITS marker for fungi.

### 2.3 RNA extraction and cDNA synthesis

Total RNA was extracted from the plant samples of *Dalbergia sissoo* showing resistance under dieback disease challenge. A 100 mg of plant material was used for RNA isolation and purification using the GeneJET Plant RNA Purification kit and RapidOut DNA Removal kit (Thermo Scientific, United States), respectively, opting for the protocol given by the manufacturer. The dried palette of each sample was dissolved in nuclease-free water and immediately used for down stress applications; the extracted RNA samples were analyzed at 260/280 nm and 260/230 nm absorbance using UV visible NANODROP (8000 Spectrophotometer, Thermo Scientific), followed by cDNA synthesis using RevertAid First Strand cDNA Synthesis Kit (Thermo Scientific, United States). The cDNA samples were diluted (20 folds) and stored (−20°C). The quantification of synthesized cDNA was determined (at 260/280 nm absorbance).

### 2.4 Primer designing

The conserved regions of the NBS-LRR class of R genes were targeted for probing the genetic elements involved in the resistance against dieback in *Dalbergia sissoo*. The degenerate primers used in DOP-rtPCR were designed based on the NBS domain. The NBS region of the NBS-LRR domain consists of different conserved domains, like kinase P-loop, kinase-2, kinase-3A, and hydrophobic GLPL motifs, which play an essential role in plant defense mechanisms (Supplementary Table S1).

### 2.5 Degenerate oligonucleotide-primed-reverse transcription PCR

The transcriptome probing of *Dalbergia sissoo* under dieback challenge was performed through degenerate oligonucleotide-primed-reverse transcription PCR (DOP-rtPCR). The PCR products were analyzed on a 1.2% high-resolution agarose gel (ACTGene). The amplicons were excised from the gel, eluted using a gel purification kit (Favor-Prep), and cloned in the TA cloning vector (pTZ57R/T); the cloned products were directly sequenced through Eurofins Genomics DNA sequencing services, United States. After sequencing, the generated sequences were trimmed (BioEdit version 7.2.6.1). The high-quality trimmed sequences were BLAST (basic local alignment search tool) in NCBI (National Center for Biotechnology Information) to check their homology to stress-responsive genetic elements. Furthermore, bioinformatics-based *in silico* analyses were performed for the structural and functional characterization of the identified sequences.

### 2.6 Scanning of conserved domains and protein fingerprints

The generated sequence was subjected to Blastx to check its homology. The ExPASy translate tool (https://web.expasy.org/translate/) was used to get translated protein sequences. The translated protein was scanned for structural motifs using the ScanProsite tool (https://prosite.expasy.org/scanprosite/). The conserved domains were identified by CD-search and CDART (Geer et al., 2002). However, protein fingerprints were scanned by the PRINTS database (http://130.88.97.239/cgi-bin/dbbrowser/fingerPRINTScan/FPScan_fam.cgi).

### 2.7 Protein localization and physicochemical characterization

The biological function of any protein is correlated to its subcellular location. Therefore, online servers CELLO v. 2.5 (Yu et al., 2006) and SignalP 5.0 (Armenteros et al., 2019) were used to predict the subcellular localization of proteins or signal peptides. The physicochemical parameters were computed using an online ExPASy tool, ProtParam (Gasteiger et al., 2005).

### 2.8 Functional annotation

The web-based tools MOTIF (https://www.genome.jp/tools/motif/) and ProtoNet (Rappoport et al., 2012) were employed to identify the conserved sites or motifs. The CATH database (Orengo et al., 1997) was used to identify superfamily and functional family predictions. Functional annotation in terms of ligand binding sites, active sites prediction was estimated through the I-TASSER web server (Yang and Zhang, 2015), and Gene Ontology (GO) (molecular function, biological process, and cellular component) was determined through COFACTOR (Zhang et al., 2017) and COACH (Yang et al., 2013).

### 2.9 Protein modeling

The secondary structures of the translated amino acid sequences of identified RGAs were predicted and annotated using the SOPMA (self-optimized prediction from multiple alignments) (Geourjon and Deleage, 1995) online server. For homology modeling analysis, an online web server, SWISS-MODEL (Waterhouse et al., 2018), and PHYRE2 (Protein Homology/analogy Recognition Engine, Version 2.0) (Kelley et al., 2015) were used. The templates with significant score values were selected. Their PDB files were used in PyMOL v. 2.4.0 for 3-D protein imaging. The predicted protein structures were validated in ProSA-web (protein structure analysis) (Wiederstein and Sippl, 2007), getting a Z-score or energy criteria. PROCHECK (Laskowski et al., 1993) was used to check quality along with stereochemistry, and TM-align (Zhang and Skolnick, 2005) was used for model superimposition with template whose molecular graphics were visualized in RasMol v.2.7.5 (Bernstein, 2009) and RasTop v. 2.2.5.

## 3 Results

### 3.1 Screening of inoculated *Dalbergia sissoo* plants

Dieback is considered a mysterious disease with no well-established or well-known etiology that leads to poor disease management. However, surveying for the *D. sissoo* plant collection, we found almost every plant affected by this disease. We generally observed that plants were resistant but also affected by dieback disease after some time. Therefore, we tried to collect the plant material that looked healthy, particularly those that stood healthy amongst the diseased plants. The healthy collected *D. sissoo* material was macro propagated under controlled conditions and maintained in a greenhouse in pots with sterilized autoclaved compost. We maintained sterile conditions before inoculation to ensure that the stress had to be due to the pathogen to be used in inoculation and not due to other factors. The collected material was screened by giving pathogen stress, and those plants that showed resistance were selected for downstream applications. Before inoculation, the healthy grown macro-propagated plant material of *D. sissoo* was probed for bacteria and fungi presence by subjecting their isolated DNA to 16S and fungal ITS DNA marker-based PCR analysis. However, we did not get any amplification in the case of both DNA markers. It showed the absence of any hidden stress by bacteria and fungi to ensure the given stress is just due to the inoculated pathogen, and the genes activated and upregulated would be against the particular stress. It was done to make the most refined identification of resistance genes or genetic elements against dieback disease. Inoculating the macro-propagated plant material of *D. sissoo*, sixteen out of 200 plants were screened as resistant or tolerant sources against dieback disease (Supplementary Table S2). Apparent dieback symptoms started one month after post-inoculation. Some plants died within a few days after symptoms appeared, while some plants died within a few months from the date of the symptoms’ appearance. However, plants showing resistance against challenge were tagged as resistant sources of *D. sissoo*. A total of sixteen plants showing resistance or tolerance under dieback challenge were subjected to degenerate oligonucleotide-primed-reverse transcription PCR (DOP-rtPCR) analysis.

### 3.2 Transcriptome-based identification of resistance gene analogs against dieback disease

Based on the NBS domain, degenerate primers were designed and synthesized to amplify resistance gene analogs (RGAs) or/candidate R genes against *D. sissoo’s* dieback disease by degenerate oligonucleotide-primed-reverse transcriptase PCR (DOP-rtPCR) analysis (Supplementary Table S1). Among these degenerate primers, dgPL-a2F/dgGL-b1R and dgPL-a2F/dgGL-b2R primer pairs gave amplifications in JSP2, NF1, and NFP2 plants of *D. sissoo*. The primer pair dgPL-a1F/dgGL-b1R gave amplification in the KPK P4 plant; the primer pair dgPL-a2F/dgGL-b2R gave amplification in the HP plant; the primer pair dgPL-a1F/dgGL-b2R gave amplification in the RKP2 plants. Amplified products were sequenced. The trimmed high-quality sequences were aligned through a multiple sequence alignment program, MAFFT (multiple alignment using fast Fourier transform). The alignment displayed three identified sequences as contig; hence, their consensus sequence was obtained using DNASTAR Lasergene v. 7.1.0 SeqMan pro (SeqManTMII). The contig sequence was designated as Ds-DbRCaG-01-Rga1. The rest were designated as Ds-DbRCaG-01-Rga2p, Ds-DbRCaG-03-Rga4p, and Ds-DbRCaG-05-Rga6p.

### 3.3 Conserved domain scanning

The translated protein sequence of the Ds-DbRCaG-01-Rga1 was subjected to CDART and CD-search web tools. The identified polynucleotide DNA sequences predicted to have encoded a polypeptide chain or a protein sequence with a signature motif (the P-loop NTPase domain belongs to the P-loop NTPase superfamily) are the attributes of disease resistance genes encoding proteins. For structural motifs’ the scanning ScanProsite tool was used that showed ATP/GTP-binding site motif A (P-loop) signature motif (GgkgqGKS) along with post-translational modification sites or PTM sites, as N-myristoylation site (MYRISTYL), Casein kinase II phosphorylation site (CK2_PHOSPHO_SITE) with phosphoserine as an intra-domain predicted feature. The phosphate-binding loop is a highly conserved motif of the nucleotide-binding site (NBS) of R genes involved in ATP/GTP binding and is a fundamental feature of ATP/GTP binding proteins (Saraste et al., 1990). However, the translated protein sequences of identified RGAs (Ds-DbRCaG-01-Rga2p, Ds-DbRCaG-03-Rga4p, and Ds-DbRCaG-05-Rga6p) showed no conserved domains upon searching through CDART and CD programs.

### 3.4 Protein fingerprint scanning

The translated protein’s fingerprint of identified RGA (Ds-DbRCaG-01-Rga1) was scanned by the PRINTS database (http://130.88.97.239/cgi-bin/dbbrowser/fingerPRINTScan/FPScan_fam.cgi). The fingerprint with the highest hit was “CSAPPISMRASE,” with an E-value of 2.5e+03. This five-element fingerprint is the cyclophilin peptidylprolyl cis-trans isomerase signature. Cyclophilins (CYPs) are members of the peptidylprolyl cis-trans isomerase family involved in catalyzing cis-trans isomerization of the peptidylprolyl bond. In plants, cyclophilin and cyclophilin-like proteins are present across all the subcellular sections. Their exact physiological role in plants is still a matter of speculation, with few exceptions. However, they have been involved in different physiological processes, including organogenesis, transcriptional regulation, hormone-signaling pathways, photosynthetic signaling pathways, stress adaptation, and defense responses (Barbosa and Park, 2019).

The translated protein sequence of Ds-DbRCaG-01-Rga2p, Ds-DbRCaG-03-Rga4p, and Ds-DbRCaG-05-Rga6p displayed no conserved domains in CDART and CD program-based searches. However, in FPScan, these deduced protein sequences predicted with protein fingerprints provided a link to their involvement in stress response or immune response. The Ds-DbRCaG-01-Rga2p is predicted with a BOMBESINR (bombesin receptor signature) fingerprint. The BOMBESINR, a six-element fingerprint, tags a signature to bombesin receptors belonging to guanine-nucleotide-binding-coupled receptors (GPCRs). The GPCRs are transmembrane receptors that work as signal transduction in response to environmental stimuli or extracellular signals. The GPCR family contains seven hydrophobic regions spanning the membrane as a C-terminal phosphorylated site in the cytoplasm and an N-terminus glycosylated site in the extracellular space. These seven transmembrane regions (helices) are linked *via* three extracellular loops and three alternative cytoplasmic loops. The six-motif signature of BOMBESINR, as a derivative from initially aligned conserved segments, highlights the aligned region, characteristic of bombesin receptors. In the six-motif region, motif-1 spans at the C terminal of the transmembrane domain-2, ushering into the first external loop, motif-2 is positioned at the N-terminus of transmembrane domain-3, motif-3 spans the 2nd external loop’ segment, motif-4 occupies the third cytoplasmic loop, motif-5 spans the third external loop’ segment, ushering into transmembrane domain-7; however, motif-6 resides at the C-terminal region. Hence, the translated sequence of Ds-DbRCaG-01-Rga2p showed homology to motif-5 and motif 6 of the BOMBESINR signature. The GPCRs are well characterized in animals and yeast, but little is known about their role in plants. However, Lu and coworkers (Lu et al., 2019) explained that GPCRs are involved in plant stress tolerance besides their involvement in growth and development.

The translated sequence of Ds-DbRCaG-03-Rga4p was predicted with the HIGHMOBLTYIY (high mobility group protein) fingerprint. High mobility group (HMG) proteins are chromosomal proteins involved in transcription regulation and nuclear localization. The HMG proteins are subdivided into three families: HMG-A/T binding, HMG-box, and HMG-nucleosome binding (Grasser et al., 2007). According to the literature, HMG-box protein regulates plant immune responses (Choi et al., 2016). The HIGHMOBLTYIY is a five-element fingerprint that gives a signature for the HMG-I/HMG-Y family. These five motifs were derived from an initial alignment of seven sequences. Motif-1 encodes the DNA binding first hook domain, motif-2 and 3 span the second hook domain, motif-4 compasses the third hook domain, and motif-5 encodes the C-terminal region rich in acidic amino acids. However, the translated sequence of Ds-DbRCaG-03-Rga4p showed homology to motif-2 and motif-3 of the HIGHMOBLTYIY signature. However, no fingerprint was predicted for Ds-DbRCaG-05-Rga6p.

### 3.5 *In silico* functional characterization

For the functional characterization of proteins, molecular weight is a crucial gauge. Therefore, the translated protein sequence of the identified RGA, Ds-DbRCaG-01-Rga1 (120 amino acid residues), was subjected to the physicochemical characterization that gave the molecular weight, 13118.30 Da, molecular formula, C591H920N146O168S9, and a predicted theoretical isoelectric point (pI) of 5.53 value. The pI value revealed it as acid. The extinction coefficient provides protein–protein and protein–ligand interactions for quantitative analysis (Prabhu et al., 2020). The computed extinction coefficient was 12950M-1 cm-1 for the predicted protein concentration in water at 280 nm. The $A_{280nm}^{0.1\%}$ was 0.987 showed reduced cysteine (Cys) residues and assumed all pairs of Cys residues formed cysteine.

The instability index (II) was computed to be 37.26, which classifies this protein as stable. Because the protein has an instability index >40, it is considered unstable and *vice versa*. However, proteins with a higher aliphatic index are considered highly thermally stable proteins. The aliphatic amino acid residues contribute to the high thermal stability in a directly proportional protein to the aliphatic index. The computed aliphatic index value of translated protein Ds-DbRCaG-01-Rga1 was 90.25, showing it is thermophilic and may resist the stressed environment. The grand average of hydropathicity (GRAVY) is also a vital physicochemical property. It illustrates the protein’s interaction with water and presents its hydrophilic or hydrophobic nature. The computed gravy score of 0.133 showed its hydrophobic nature because positively rated proteins are more hydrophobic (Kyte and Doolittle, 1982).

The physicochemical properties of the deduced amino acid sequences of Ds-DbRCaG-01-Rga2p, Ds-DbRCaG-03-Rga4p, and Ds-DbRCaG-05-Rga6p were also computed. The aliphatic index of Ds-DbRCaG-01-Rga2p and Ds-DbRCaG-03-Rga4p was 100.85 and 93.07, respectively, showing their thermophilic nature. However, the aliphatic index of Ds-DbRCaG-05-Rga6p was 71.33, which is comparatively lower than Ds-DbRCaG-01-Rga1, Ds-DbRCaG-01-Rga2p, and Ds-DbRCaG-03-Rga4p, but is toward the higher side as well.

A signal peptide predicts the target site to which that protein would be transported. Information about its localization is also vital in determining the protein’s function. We predicted the cellular localization of the Ds-DbRCaG-01-Rga1 protein using the CELLO2GO web server. It predicted the cytoplasmic localization of Ds-DbRCaG-01-Rga1 with a significant score (1.237), followed by its localization in the chloroplast, scoring a 1.084 value. The Ds-DbRCaG-01-Rga2p and Ds-DbRCaG-05-Rga6p were predicted with extracellular localization. The Pathogenesis-related proteins of the tobacco (NtPR1) and Arabidopsis-related proteins (AtPR1) were reported to be localized in extracellular space (Vigers et al., 1992; Pecenkova et al., 2017). Furthermore, the localization of pathogenesis-related protein PR10.2 of *Plasmopara viticola* was predicted in subcellular compartments, i.e., the nucleus, cytoplasm, and chloroplast nuclear region (He et al., 2013).

### 3.6 *In silico* functional annotation

Ds-DbRCaG-01-Rga1 showed a conserved domain in CDART and CD-search tools. Different web-based tools were used for its *in silico* functional annotation, including MOTIF, CATH, COFACTOR, COACH, and I-TASSER. The MOTIF search tool displayed the results based on NCBI-CDD and Pfam databases. It represented the prediction of different motifs given in Table 1. However, the significant hits based on the NCBI-CDD database search characterized the translated protein sequence of RGA as ribulose bisphosphate carboxylase/oxygenase activase-RuBisCO activase (Rca) and Torsin; however, Pfam predicted the presence of Torsin and AAA+ (ATPase family associated with various cellular activities). These results supported that the translated protein sequence of Ds-DbRCaG-01-Rga1 is a disease resistance protein because “Torsins” are essential, disease-relevant AAA+ (ATPases associated with various cellular activities) proteins (Chase et al., 2017). Similarly, Rubisco activase (Rca) is a molecular chaperone or AAA + chaperone (Hayer-Hartl and Hartl, 2020), and molecular chaperons are associated with rescuing the cell in a stressed environment and contributing to plant immunity (Park and Seo, 2015). The ProtoNet tool also predicted the similarity of the identified putative disease resistance gene(s) sequence to the Rubisco activase encoding gene (Table 1) with GO molecular function and ATP binding.

**TABLE 1:** Functional motifs of Ds-DbRCaG-01-Rga1 predicted through Motif prediction tools, ProtoNet, and MOTIF.

ProtoNet	MOTIF	
	NCBI-CDD-based search with E-value	Pfam-based search with E-value
Ribulose bisphosphate carboxylase/oxygenase activase	• Ribulose bisphosphate carboxylase/oxygenase activase -RuBisCO activase (4e-70)	• Torsin (6.3e-05)
	• Torsin (7e-04)	• ATPase family associated with various cellular activities (AAA) (0.084)
	• AAA, ATPase family, associated with various cellular activities (0.001)	
	• Adenylate kinase family protein (0.032)	
	• Dna A regulatory inactivator Had (0.094)	
	• Adenylate_kinase_isoenzyme_1, adenylate kinase, isozyme 1 subfamily (0.13)	
	• AAA domain (0.41)	

The superfamily and functional family of translated protein Ds-DbRCaG-01-Rga1 were identified through the CATH database, revealing that it belongs to the superfamily P-loop containing nucleotide triphosphate hydrolases and the functional family Ribulose bisphosphate carboxylase/oxygenase activase (Rubisco activase, Rca). These results also supported it as a putative disease resistance protein because the P-loop is the signature motif of disease-resistant proteins in plants. Hence, the identified differentially expressed gene sequence of *Dalbergia sissoo* under dieback challenge was submitted to NCBI with the GenBank accession number MW533149.

Function annotation is a crucial criterion for the characterization of a protein. The COACH Meta server, based on I-TASSER structure prediction, determined the functional annotation of the translated protein of an identified disease resistance gene sequence or RGA. These databases predicted 41Phe (F), 42Tyr (Y), 44Ala (A), 47Phe (F), 77Lys (K), 78Gly (G), 80Gly (G), 81Lys (K), 82Ser (S), and 83Phe (F), as protein-ligand binding sites (Supplementary Figure S1) based on a high confidence score (C-score) that relates to more reliable prediction. The ligand-binding site residues, 77Lys (K), 78Gly (G), 80Gly (G), 81Lys (K), and 82Ser (S), are amino acid residues of the P-loop domain. The active site residue predicted using I-TASSER was 86Glu (E), as glutamate (E) residue is a characteristic feature of active kinases (Huse and Kuriyan, 2002). The Gene Ontology (GO) reconnoitered through the COFACTOR server represented its predominant role in response to stimuli and stresses (Table 2).

**TABLE 2:** *In silico* functional annotation of Ds-DbRCaG-01-Rga1 predicted through COFACTOR and COACH web-based tools.

Biological process (BP)	Molecular function (MF)		
GO term	Description with CscoreGO	GO term	Description with CscoreGO
GO:0050896	Response to stimulus (0.96)	GO:0005524	ATP binding (0.96)
GO:0009628	Response to abiotic stimulus (0.95)	GO:0003824	Catalytic activity (0.81)
GO:0006950	Response to stress (0.95)	GO:0016787	Hydrolase activity (0.76)
GO:0009266	Response to temperature stimulus (0.94)	GO:0017111	Nucleoside-triphosphatase activity (0.70)
GO:0044699	Single-organism process (0.81)	GO:0016887	ATPase activity (0.69)
GO:0008152	Metabolic process (0.76)		
GO:0009408	Response to heat (0.63)		
GO:0009987	Cellular process (0.56)		
GO:0044710	Single-organism metabolic process (0.55)		

CscoreGO is the confidence score of predicted GO terms. CscoreGO, values range between [0–1], where a higher value indicates better confidence in predicting the function using the template.

### 3.7 Annotation of protein secondary structure

As no conserved domains were predicted in Ds-DbRCaG-01-Rga2p, Ds-DbRCaG-03-Rga4p, and Ds-DbRCaG-05-Rga6p, through CD-search and CDART; therefore, these RGAs were subjected to structure prediction to unravel their role in disease resistance. The SOPMA program was executed to predict their secondary structure. The α-helix (Hh), random coil (Cc), β-turn (Tt), and extended strand (Ee) percentiles were observed in the translated protein sequences of these RGAs. The 20.34% Hh and 20.34% Ee with a significant share of Cc at 50.50% were predicted in Ds-DbRCaG-01-Rga2p′s translated protein sequence. The Ds-DbRCaG-03-Rga4p′s translated protein sequence was predicted with 17.54% Hh, 25.44% Ee, and 7.02% Tt, with a significant share of Cc at 50%. The Ds-DbRCaG-05-Rga6p′s translated protein sequence was also predicted with 18.75% Ee and 6.25% Tt with a significant share of Cc at 75% secondary features.

### 3.8 Homology modeling and 3D imaging

The 3D model of Ds-DbRCaG-01-Rga2p displayed homology to the template 1X6I (crystal structure of ygfY from *Escherichia coli*). A total of thirty-one amino acid residues of Ds-DbRCaG-01-Rga2p (9–39) have been modeled with template 1X6I residues (50–80) with 7.26% confidence and 13% sequence identity. The aligned region of the template confirmed the succinate dehydrogenase_SDH5 superfamily domain. Succinate dehydrogenase (SDH) is an enzyme involved in mitochondrial reactive oxygen species (ROS), while SDH is involved in enhancing the production of ROS in any plant stress. ROS production is a crucial indicator of the plant stress response. Likewise, the FAD (flavin adenine dinucleotide) cofactor attachment to SDH1 through the delivery vehicles, SDH5 or SDHAF2 (Eletsky et al., 2012), mediates the production of fumarate from succinate in the tricarboxylic acid cycle (Krebs cycle). Moreover, if the plant suffers from stress, SDH upregulates the expression of stress-related genes (Jardim-Messeder et al., 2015). The enhanced production of salicylic acid also correlates with mitochondrial ROS, ultimately SDH (Belt et al., 2017). SDH has subunits, SDH1-SDH5, though SDH5 has been newly identified in prokaryotes and eukaryotes. Even its homologous, SDHAF2 (succinate dehydrogenase assembly factor 2), has also been identified in *Arabidopsis thaliana* (Huang et al., 2013). SDH5 was identified to be involved in the flavinylation process.

Ds-DbRCaG-03-Rga4p showed maximum homology with a significant hit to template 1GC6 (crystal structure of the radixin FERM domain complexed with inositol-(1,4,5)-triphosphate). A total of fifteen amino acid residues of Ds-DbRCaG-03-Rga4p (28–42) have been modeled with template 1GC6 residues (275–289) with 28% confidence and 13.33% sequence identity. The aligned region of the template revealed the pleckstrin homology (PH) like domain. Several proteins involved in cellular signal transmission, membrane transport, phospholipid modification, and cytoskeletal regulation are characterized as having a PH domain (Blomberg and Nilges, 1997; Rebecchi and Scarlata, 1998; Lemmon et al., 2002; Huang et al., 2013). The PH domains are lipid or phospholipid-binding domains that facilitate membrane localization (Maffucci and Falasca, 2001). The PH domain association in lipid binding provides the association of PH domain-containing proteins with lipid signaling that enhances plant disease resistance (Tang et al., 2005).

Ds-DbRCaG-05-Rga6p showed homology by significantly hitting the template 6I4H (crystal structure of plasmodium falciparum actin I (F54Y mutant) in the Ca-ATP state). A total of twenty-nine amino acid residues of Ds-DbRCaG-05-Rga6 (1–29) have been modeled with template 6I4H residues (7–35) with 31% sequence similarity and 17.24% sequence identity. However, the template’s aligned region confirmed the nucleotide-binding domain of the sugar kinase/HSP70/actin superfamily. As the superfamily members of the sugar kinases, actin, and heat shock-related proteins, they showed structural homology in 3D folding but differed in their function. However, these proteins are characterized by ATP hydrolysis or phosphotransferase (Hurley, 1996). The sugar kinases are involved in metabolic regulation, as does the ATP-dependent phosphorylation of sugars. HSP70 is a well-known cellular protein folding chaperone with a specialized ATP-binding domain. Actin is a cellular filament that plays a central role in the cytoskeleton structure. All three proteins have a specialized nucleotide-binding region in a similar position and conformation.

**FIGURE 1:**
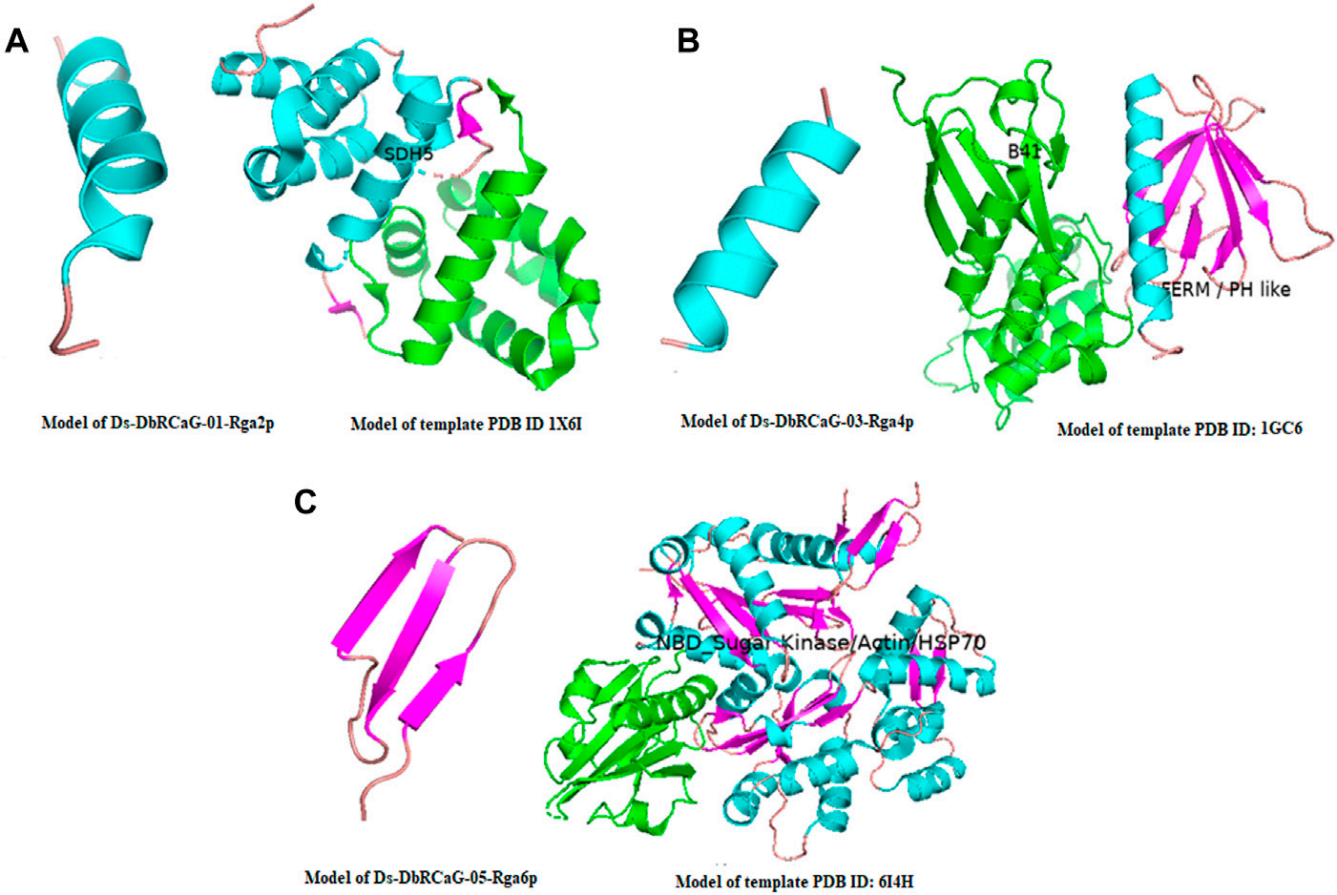
3-D imaging of Ds-DbRCaG-01-Rga2p, Ds-DbRCaG-03-Rga4p, and Ds-DbRCaG-05-Rga6p. The homology zone of the template model and the model of predicated protein structure of each identified RGA showing with same colors: **(A)** predicted protein structure of Ds-DbRCaG-01-Rga2p, **(B)** predicted protein structure of Ds-DbRCaG-03-Rga4p, and **(C)** predicted protein structure of Ds-DbRCaG-05-Rga6p. The secondary models of template proteins were retrieved from the RCSB Protein Data Base, https://www.rcsb.org/

The crystalline structure of the template 1X6I chain A showed the SDH5 domain. The model 1X6I template is displayed as a helical bundle of multiple helices joined by loops. The predicted model of Ds-DbRCaG-01-Rga2p’s translated protein displayed structural homology to the part of the SDH5 domain of the template model, as shown in Figure 1A; the homology zone between the template model and the predicted translated protein of RGA share the same color. The crystalline structure of template 1GC6 showed two domains: (1) the N-terminal B41 domain and (2) a C-terminal FERM or PH-like domain connected with the loop. The predicted model of Ds-DbRCaG-03-Rga4p’s translated protein showed structural homology to a PH-like domain possessing perpendicular antiparallel β strands and one helix (Figure 1B). The crystalline structure of the template 6I4H chain A displayed the NBD domain of sugar kinase/HSP70/actin with a conserved core structure, βββαβαβα. The turns between β1 and β2 majorly contribute to the nucleotide-binding site. The predicted model of Ds-DbRCaG-05-Rga6p′s translated protein showed structural homology at different regions of the NBD domain for three β-pleated sheets (predicted model of Ds-DbRCaG-05-Rga6p) joined *via* loops, as shown in Figure 1C.

### 3.9 3D model validation

**FIGURE 2:**
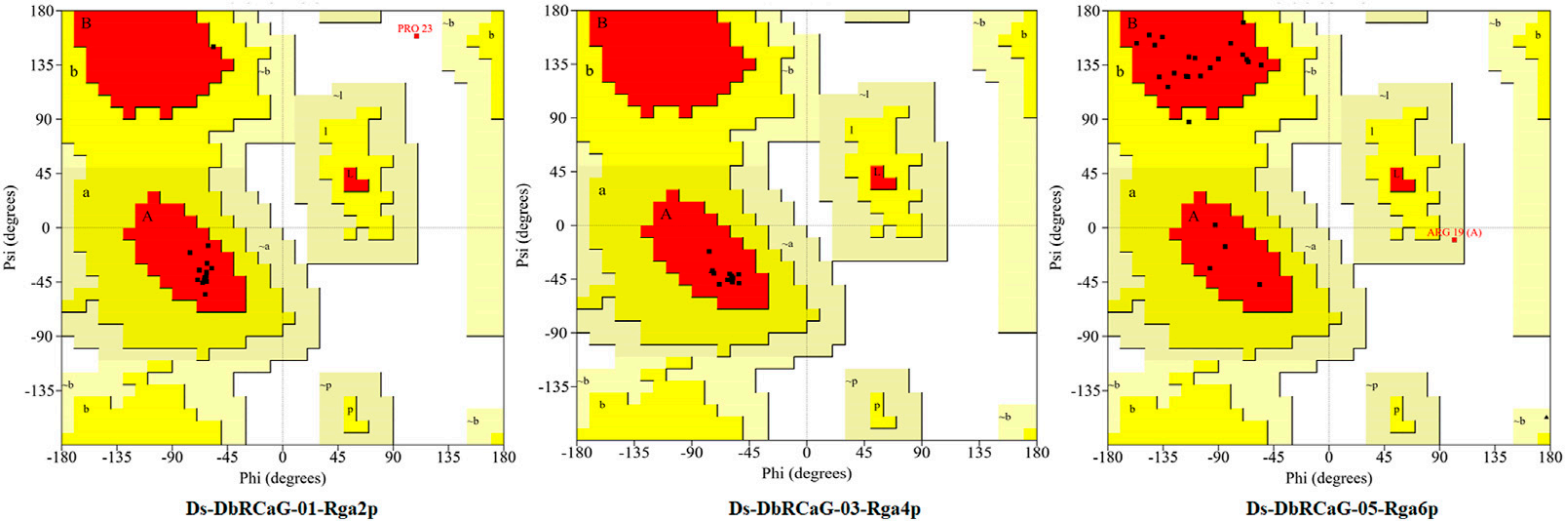
Ramachandran plot of Ds-DbRCaG-01-Rga2p, Ds-DbRCaG-03-Rga4p, and Ds-DbRCaG-05-Rga6p. Their most favored region is red, additionally allowed in yellow, generously allowed in light yellow, and disallowed regions are indicated in white fields.

**FIGURE 3:**
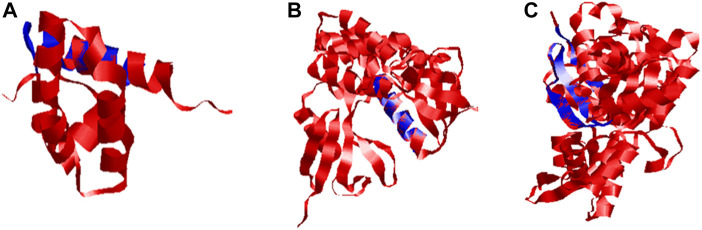
Superposed ribbon structure of predicted proteins models of Ds-DbRCaG-01-Rga2p, Ds-DbRCaG-03-Rga4p, and Ds-DbRCaG-05-Rga6p with their respective template regions. Blue indicates identified RGAs predicted proteins, while red indicates the template protein. **(A)** Ds-DbRCaG-02-Rga2 superposed to model 1X6I, **(B)** Ds-DbRCaG-03-Rga4p superposed to model 1GC6, and **(C)** Ds-DbRCaG-05-Rga6p superposed to model 6I4H.

The quality of 3D models was determined by generating Ramachandran plots. The plot consisted of amino acid residues found in Phi (φ) and Psi (ψ) angles based on the graphical data representation. For Ds-DbRCaG-01-Rga2p and Ds-DbRCaG-03-Rga4p, the highest value model was observed with 100% residues in the most favored region. The model validation study of Ds-DbRCaG-05-Rga6p indicated 90.9% residues in the most favored region, 4.5% in the additional allowed region, and 4.5% in the generously allowed region. In general, the value ≥ 90% of residues in the most favored region is crucial to predict that the model is of good quality (Figure 2). The calculated Z-scores for predicted models of Ds-DbRCaG-01-Rga2p, Ds-DbRCaG-03-Rga4p, and Ds-DbRCaG-05-Rga6p were -1.43, -0.63, and -0.18, respectively, validating the models to be satisfactory. The superimposition of the models with the templates was analyzed by computing TM scores and RMSD (root mean square deviation) values. The TM score of Ds-DbRCaG-01-Rga2p, Ds-DbRCaG-03-Rga4p, and Ds-DbRCaG-05-Rga6p was 0.15016, 0.05050, and 0.08237, respectively, unraveling the random structural similarity of superimposed models with their templates. In comparison, the RMSD values of Ds-DbRCaG-01-Rga2p, Ds-DbRCaG-03-Rga4p, and Ds-DbRCaG-05-Rga6p with their respected templates were 0.97 Å, 0.06 Å, and 0.11 Å, respectively. The RMSD value scales the similarity between two atomic coordinates in a superimposed position. The lower the RMSD value (< 2), the higher the accuracy of the results (Kirchner and Guntert, 2011). The estimated RMSD values of models imparted confidence that residues were well superimposed. The predicted models were folded exactly in the same manner as their respective templates. The superposed full atom structures of the whole chain are given in Figure 3. The *in silico* prediction of the expressome of *Dalbergia sissoo* under dieback disease stress revealed the role of identified RGAs in disease and stress resistance.

## 4 Discussion

Plants have an innate immune system against pathogens. Upon infecting plants, pathogens produce pathogen-associated molecular patterns (PAMPs), and plants detect them by transmembrane pattern recognition receptors, which leads to the activation of signaling pathways and is known as PAMP-triggered immunity (Dodds and Rathjen, 2010). The strong invaders suppress this immunity by secreting effector proteins or effectors into plant cells to arrest signaling pathways (Kamoun, 2006). In response to effectors, plants generate intracellular immune receptors to recognize the effectors and develop effector-triggered immunity. This immunity establishes a hypertensive response resulting from programmed activation of localized cell death (Jones and Dangl, 2006). The immune receptors encoded by R genes are particular. These intracellular R proteins have N-terminal nucleotide-binding sites (NBS) and a C-terminal leucine-rich repeat (LRR). The R proteins recognize and interact with their corresponding effectors through the N-terminal NBS domain (Dangl and Jones, 2001; Mucyn et al., 2006; Burch-Smith et al., 2007), which indicates the recognition specificity of specific R genes to their specific effectors (Luck et al., 2000). The N-terminal NBS domain of the NBS-LRR class of R proteins is projected for effector recognition (Ade et al., 2007).

Therefore, in this study, to identify resistance gene analogs (candidate resistance gene sequences) in *D. sissoo* against dieback disease, the nucleotide-binding site (NBS) of the NBS-LRR class of R genes was used to probe the transcriptome of *D. sissoo*. The NBS region of the NBS-LRR domain consists of different conserved domains, like kinase P-loop, kinase-2, kinase-3A, and hydrophobic GLPL motifs (McHale et al., 2006), which play an essential role in plant defense mechanisms. The Blast tool revealed the differentially expressed identified DNA sequence upregulated under dieback stress has homology to the P-loop NTPase superfamily with a significant hit. The P-loop domain has been reported to be a part of NBS-LRR containing RGAs in different plant species like rice, sugar beet, maize, and coconut and is considered a characteristic domain of disease resistance proteins in plants (Tian et al., 2004; Wenkai et al., 2006; Rachana et al., 2016).

The MOTIF web-based tool characterized the RGA translated protein sequence as ribulose bisphosphate carboxylase/oxygenase activase-RuBisCO activase (Rca) and Torsin; however, Pfam predicted the presence of Torsin and AAA+ (ATPase family associated with various cellular activities). This characterization revealed the translated protein sequence of the identified RGA as a disease resistance protein because “Torsins” are essential, disease-relevant AAA+ (ATPases associated with various cellular activities) proteins (Chase et al., 2017). Correspondingly, Rubisco activase (Rca), as a molecular chaperone (AAA + chaperone), is associated with extricating the cell in a stressed environment and contributing to plant immunity (Park and Seo, 2015; Hayer-Hartl and Hartl, 2020). Similarly, the functional annotation of the translated protein sequence of the identified RGA using COFACTOR, COACH, and I-TASSER bioinformatics tools reconnoitered its predominant role in response to stresses.

The translated sequences of Ds-DbRCaG-01-Rga2p, Ds-DbRCaG-03-Rga4p, and Ds-DbRCaG-05-Rga6p were found to have no-match of conserved domains from databases for conserved domains. Predicted protein fingerprints search displayed bombesin receptor signature (BOMBESINR) for Ds-DbRCaG-01-Rga2p that showed it as transmembrane receptors; guanine-nucleotide-binding protein-coupled receptors (GPCRs) reported to be involved in stress tolerance in plants (Lu et al., 2019). Ds-DbRCaG-03-Rga4p is predicted to possess a fingerprint of a high mobility group protein (HIGHMOBLTYIY) that contributes to regulating plant immune response (Choi et al., 2016).

Using online web servers, we performed the structural characterization of identified RGAs. The Ds-DbRCaG-01-Rga2p showed homology to the template PDB ID: 1X6I. The alignment region confirmed the succinate dehydrogenase_SDH5 domain, involved in producing reactive oxygen species (ROS) and upregulating stress-responsive genes (Jardim-Messeder et al., 2015; Belt et al., 2017). The Ds-DbRCaG-03-Rga4p showed significant homology to template PDB ID: 1GC6. The alignment region between the template model and the Ds-DbRCaG-03-Rga4p predicted protein model validated the homology with lipid or-binding pleckstrin homology (PH), strongly associated with disease resistance (Tang et al., 2005). The structural homology of the predicted protein model of Ds-DbRCaG-05-Rga6p with the nucleotide-binding domain of sugar kinase/HSP70/actin domain of template model PDB ID: 6I4H revealed strong support for its putative role in stress resistance (Hurley, 1996). The molecular and bioinformatics-based analyses predicted the presence of motifs including ATP/GTP-binding site motif A (P-loop NTPase domain), GLPL domain, casein kinase II phosphorylation site, and N-myristoylation site, etc. that are the attributes of proteins encoded by disease resistance genes. The physicochemical attributes of identified resistance gene analogs, subcellular localization, predicted protein fingerprints, *in silico* functional annotation, and predicted protein structure proved their role in disease and stress resistance.

## Data Availability

The datasets presented in this study can be found in online repositories. The names of the repository/repositories and accession number(s) can be found at: https://www.ncbi.nlm.nih.gov/, MW533149.
