# Supplementary material for: Identification of resistance gene analogs of the NBS-LRR family through transcriptome probing and in silico prediction of the expressome of Dalbergia sissoo under dieback disease stress
Source: Front Genet. 2022 Oct 7;13:1036029. doi: 10.3389/fgene.2022.1036029 (PMC9585183; doi:10.3389/fgene.2022.1036029)
Supplement: Supplementary file 1 [file DataSheet1.docx]

**
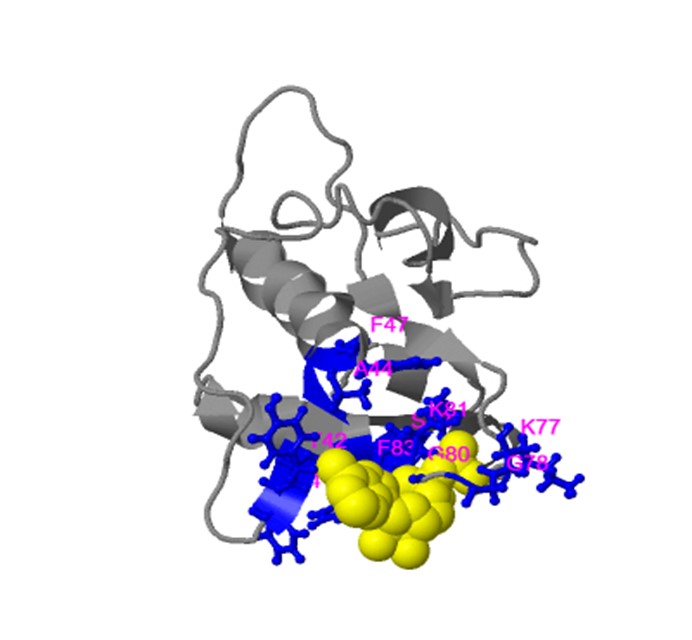
**

**Figure S1** Protein-ligand binding sites of identified putative resistance gene sequences or RGAs in shisham upregulated against dieback disease, predicted through COACH Meta server based on I-TASSER structure prediction

**Table S1:** Degenerate primers of NBS domain used to recover putative resistance gene sequences or RGAs in shisham upregulated against dieback disease.

| *Degenerate primer* | *Primer sequence (5′-3′)* | *References* |
| --- | --- | --- |
| dgPL-a1F | GGNGGNRTNGGNAAGACGAC | (Noir *et al*., 2001) |
| dgPL-a2F | GGNGGNRTIGGIAARACIAC | (Sun *et al*., 2010) |
| dgPL-a3F | GGIGGIGTIGGIAAIACIAC | (Leister *et al*., 1996) |
| dgPL-a4F | TGSSRGGHWYRGGBAAAACTAC | (Zhang *et al*., 2008) |
| dgPL-a5F | GGTGGGGTTGGGAAGACAACG | (Leister *et al*., 1996) |
| dgGL-b1R | GAGGGCTAAAGGAAGGCC | (Deng *et al*., 2000) |
| dgGL-b2R | IAGIGCIAGIGGIAGICC | (Leister *et al*., 1996) |
| dgGL-b3R | AAGIGCTAAGIGGIAAGICC | (Peraza-Echeverria *et al*., 2008) |
| dgGL-b4R | HRCWARAGGVARCCCTYBACA | **(**Naresh *et al*., 2017). |
| dgGL-b5R | GAGGGCNARNGGNAAICC | (Noir *et al*., 2001) |

**Table S2:** *Dalbergia sissoo* screened plant material shown resistance against dieback disease

| **S No.** | **Plant Code** | **Location** | **Status** | **S No.** | **Plant Code** | **Location** | **Status** |
| --- | --- | --- | --- | --- | --- | --- | --- |
| 1 | FP1 | Faisalabad | Tolerant | 9 | HP1 | Hyderabad | Resistant |
| 2 | CP1 | Chichawatni | Tolerant | 10 | HP2 | Hyderabad | Resistant |
| 3 | TP1 | Toba Tek Singh | Tolerant | 11 | KPP1 | Khairpur | Resistant |
| 4 | TP3 | Toba Tek Singh | Tolerant | 12 | KPP2 | Khairpur | Resistant |
| 5 | RKP2 | Rahim Yar Khan | Tolerant | 13 | NFP1 | Naushahro Feroze | Resistant |
| 6 | RKP3 | Rahim Yar Khan | Resistant | 14 | NFP2 | Naushahro Feroze | Resistant |
| 7 | BLP1 | Jaffarabad | Tolerant | 15 | JSP1 | Jamshoro | Tolerant |
| 8 | KPKP4 | Khyber Pakhtunkhwa | Resistant | 16 | JSP2 | Jamshoro | Resistant |
